# Loss of the deubiquitylase BAP1 alters class I histone deacetylase expression and sensitivity of mesothelioma cells to HDAC inhibitors

**DOI:** 10.18632/oncotarget.3765

**Published:** 2015-04-24

**Authors:** Joseph J. Sacco, Jenna Kenyani, Zohra Butt, Rachel Carter, Hui Yi Chew, Liam P. Cheeseman, Sarah Darling, Michael Denny, Sylvie Urbé, Michael J. Clague, Judy M. Coulson

**Affiliations:** ^1^ Cellular & Molecular Physiology, Institute of Translational Medicine, University of Liverpool, Liverpool, UK; ^2^ Current address: Cancer Stem Cell Biology, Agency for Science Technology and Research, Genome Institute of Singapore, Singapore; ^3^ Current address: MRC Laboratory of Molecular Biology, Cambridge, UK

**Keywords:** histone deacetylase 2, BRCA1-associated protein 1, MPM, vorinostat, stratified medicine

## Abstract

Histone deacetylases are important targets for cancer therapeutics, but their regulation is poorly understood. Our data show coordinated transcription of HDAC1 and HDAC2 in lung cancer cell lines, but suggest HDAC2 protein expression is cell-context specific. Through an unbiased siRNA screen we found that BRCA1-associated protein 1 (BAP1) regulates their expression, with HDAC2 reduced and HDAC1 increased in BAP1 depleted cells. BAP1 loss-of-function is increasingly reported in cancers including thoracic malignancies, with frequent mutation in malignant pleural mesothelioma. Endogenous HDAC2 directly correlates with BAP1 across a panel of lung cancer cell lines, and is downregulated in mesothelioma cell lines with genetic BAP1 inactivation. We find that BAP1 regulates HDAC2 by increasing transcript abundance, rather than opposing its ubiquitylation. Importantly, although total cellular HDAC activity is unaffected by transient depletion of HDAC2 or of BAP1 due to HDAC1 compensation, this isoenzyme imbalance sensitizes MSTO-211H cells to HDAC inhibitors. However, other established mesothelioma cell lines with low endogenous HDAC2 have adapted to become more resistant to HDAC inhibition. Our work establishes a mechanism by which BAP1 loss alters sensitivity of cancer cells to HDAC inhibitors. Assessment of BAP1 and HDAC expression may ultimately help identify patients likely to respond to HDAC inhibitors.

## INTRODUCTION

The histone deacetylases (HDACs) are an ancient and highly conserved family of enzymes that catalyze removal of acetyl groups from lysine residues, antagonizing the effects of histone acetyl-transferases (HATs). The HDACs play key roles in epigenetic modulation of gene expression through their activity towards histones. However, a considerable proportion of the proteome is subject to reversible acetylation [1] and, as HDACs have activity towards a plethora of protein substrates, they are more accurately termed lysine deacetylases. They comprise two sub-families: eleven zinc-dependent isoenzymes that are divided into class I (HDAC1, HDAC2, HDAC3, HDAC8), class IIa (HDAC4, HDAC5, HDAC7, HDAC9), class IIb (HDAC6, HDAC10) and class IV (HDAC11); plus seven NAD^+^-dependent sirtuins that form a functionally distinct class III (SIRT1-SIRT7) [2]. This multiplicity of HDACs reflects their diverse and tissue-specific functions.

Class I HDACs are widely expressed and, within this class, HDAC1 and HDAC2 are most closely related. They have a near identical gene structure indicative of duplication from a common ancestor, and retain 86% amino acid sequence identity. HDAC1 and HDAC2 can homo- and hetero-dimerize, but are commonly found together in multi-protein complexes on which their catalytic activity depends. HDAC1 and HDAC2 are predominantly nuclear, usually associated with chromatin and play key transcriptional roles, although they have also been implicated in splicing, mitosis, meiosis, DNA replication and DNA repair (reviewed in [3]).

Controversy has raged over the redundancy or isoenzyme specificity of HDAC1 and HDAC2, with loss-of-function studies in mice delivering important yet sometimes conflicting data (reviewed in [3]). In germline knockouts, HDAC1 is embryonic lethal before E10.5, but HDAC2 null mice can survive until and beyond the perinatal period, perhaps suggesting independent functions. However, conditional isoenzyme knockouts have less severe phenotypes, and a compensatory mechanism is commonly seen, where deletion of HDAC1 results in increased expression of HDAC2 protein, and vice versa. Combined conditional ablation of HDAC1 and HDAC2 has dramatic effects on proliferation, survival, and/or differentiation in most tissues and cell types. Ultimately, it has required models with deletion of 3 out of the 4 HDAC1 and HDAC2 alleles to allow elucidation of the overlapping but specific roles for HDAC1 and HDAC2 during development.

Importantly, HDAC1 and HDAC2 are dysregulated in many human diseases including diabetes, asthma, neurological disorders and cancer. In breast or osteoscarcoma cells HDAC1, but not HDAC2, was required for proliferation and its depletion led to cell cycle arrest and apoptosis [4]. Intriguingly though, HDAC2 is specifically degraded in airway disease through stress-responsive post-translational modifications (PTMs) triggered by cigarette smoke [5]. Both HDAC1 and HDAC2 are known to be phosphorylated, acetylated, ubiquitylated, SUMOylated, nitrosylated and alkylated (reviewed in [6]). Isoenzyme specific PTMs, particularly within the less well conserved C-terminal, may contribute to the context-specific redundancy or independent activities of HDAC1 and HDAC2 [6]. In the setting of reversible ubiquitylation, alternative ubiquitin E3 ligases have been identified for HDAC1 [7, 8] and HDAC2 [9, 10], which either modulate their transcriptional activity or target them for proteasomal degradation, but no deubiquitylases (DUBs) have yet been identified that antagonize these functions.

Given their deregulation in cancer HDAC1 and HDAC2 are important therapeutic targets. First generation pan-HDAC inhibitors, such as vorinostat (SAHA), bind the active site Zn^2+^ ion critical for the function of class I, II and IV HDACS. Their anti-tumor activity is largely attributed to selective induction of cell cycle arrest and/or apoptosis in cancer cells [2]. Vorinostat was the first HDAC inhibitor to be licensed, for use in treatment of cutaneous T-cell lymphoma [11]. However vorinostat has shown limited efficacy in single agent trials for solid tumors [12]. Accumulating evidence that specific HDAC isoenzymes deacetylate distinct target proteins, and are differentially expressed in cancers, argues for the use of more specific HDAC inhibitors. Class I or class II selective inhibitors are available, and isotype-selective inhibitors are in development [13]. Identifying cellular targets that sensitize to HDAC inhibition may enable stratification of patients within trials.

We set out to investigate the co-expression of HDAC1 and HDAC2 in lung cancer cell lines and to use an unbiased siRNA screen to identify DUBs that may regulate HDAC1 and/or HDAC2 expression. We found that depletion of the tumor suppressor BAP1 reduced HDAC2 expression, acting at the level of transcription rather than ubiquitylation. This provides a clear mechanism by which BAP1 loss-of-function alters cancer cell sensitivity to specific HDAC inhibitors.

## RESULTS

**Figure 1:**
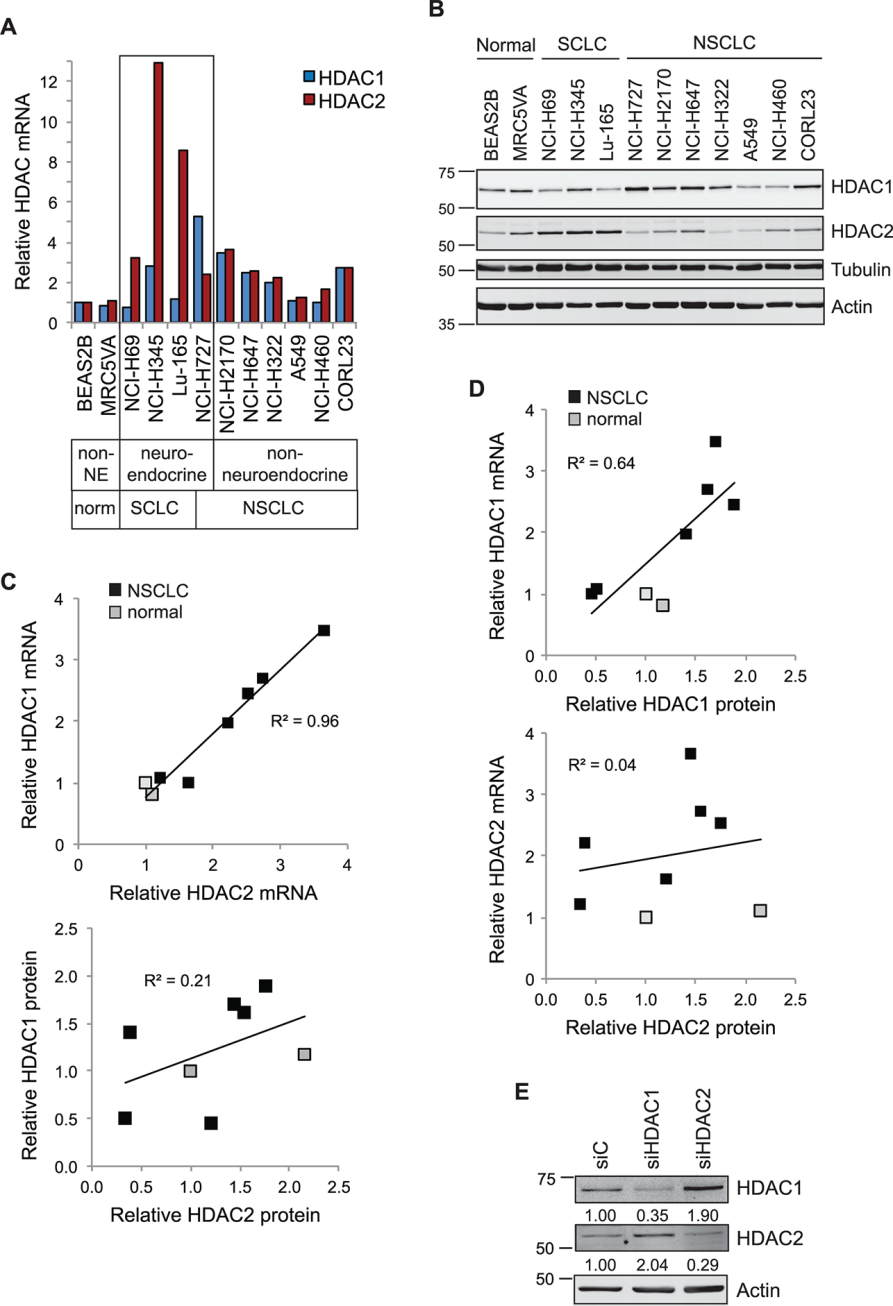
Interdependency of HDAC1 and HDAC2 expression in lung cancer cell lines **A.** HDAC1 and HDAC2 transcript expression in lung cancer cells. RNA extracted from a panel of normal lung and lung cancer cell lines was assessed for HDAC transcript expression by qRT-PCR. Cq values for test genes were normalized to actin and expressed as 2^−[ΔΔCq]^ relative to the BEAS2B cell line. **B.** Co-extracted proteins from the same cell lines were immunoblotted for HDAC1 and HDAC2. For correlation in subsequent panels, HDAC protein levels were quantified using Odyssey analysis software, normalized to tubulin and shown relative to the BEAS2B cell line. **C.** HDAC1 and HDAC2 transcripts (top), but not proteins (below), exhibit strong positive correlation in the NSCLC and normal lung cell lines. **D.** HDAC1 mRNA (top), but not HDAC2 mRNA (below), is a good predictor of protein expression. Scatter plots represent quantification of protein (x-axis) plotted against that of mRNA (y-axis). **E.** Depletion of HDAC1 or HDAC2 leads to isoenzyme compensation. A549 cells were transfected with 40 nM siRNAs as indicated. Whole cell protein extracts were prepared 72 hr later and equivalent amounts immunoblotted. Expression of each HDAC was normalized to actin and is indicated relative to the siC control.

We initially investigated the interdependence of HDAC1 and HDAC2 expression, and evaluated the relative contribution of transcriptional or post-transcriptional regulation towards their protein level expression. To this end, we simultaneously extracted total RNA and cellular protein from a panel of lung cancer cell lines for quantitative RT-PCR and immunoblotting (Figure 1A and 1B). The panel comprised cell lines derived from normal bronchial epithelium or fibroblasts, together with three small cell lung cancer (SCLC), one carcinoid and six non-small cell lung cancer (NSCLC) cell lines. We saw very tight correlation between the transcript levels of HDAC1 and HDAC2 across the eight non-neuroendocrine cell lines, with increased expression in NSCLC relative to normal lung (Figure 1A and 1C), suggesting that transcription of HDAC1 and HDAC2 may be co-regulated. However, expression of HDAC1 and HDAC2 were uncoupled in neuroendocrine cell lines, so that in each case one of the isoenzyme transcripts was markedly elevated relative to the other: HDAC2 in SCLC (NCI-H69, NCI-H345 and Lu-165) and HDAC1 in the NCI-H727 carcinoid cell line (Figure 1A). In non-neuroendocrine cells, HDAC1 mRNA was a good predictor for the HDAC1 protein expression level, but this was not true for HDAC2 (Figure 1D). Thus, in a given cellular context, the level of HDAC2 may be largely dependent on post-transcriptional, translational or post-translational regulatory mechanisms. As compensatory responses in protein expression are reported between the HDAC1 and HDAC2 isoenzymes in conditional knockout mice [3], and on siRNA depletion in osteosarcoma and breast cancer cell lines [4], we tested if this was also the case in A549 lung cancer cells. As predicted, we saw increased HDAC2 protein in HDAC1 depleted cells, and increased HDAC1 protein in HDAC2 depleted cells (Figure 1E).

**Figure 2:**
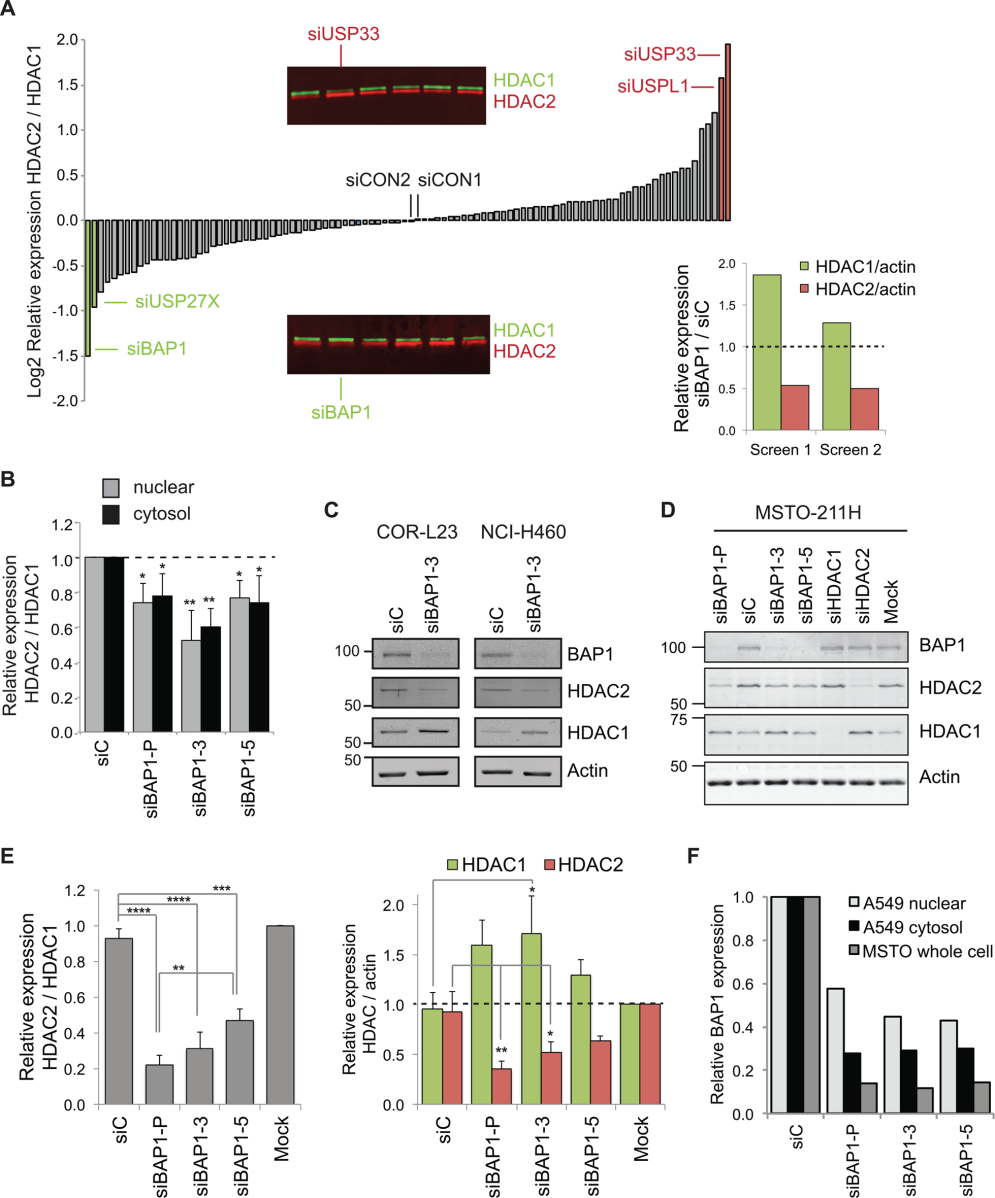
BAP1 regulates the balance of HDAC2 and HDAC1 expression **A.** DUB siRNA library screen identifies BAP1 as a candidate regulator of class I HDACs. 72 hr after transfection with 40 nM siRNA pools against 92 human DUBs, their effect on HDAC1 and HDAC2 expression was screened by immunoblotting of nuclear extracts from A549 lung cancer cells. The main histogram shows mean log(2) values for HDAC2 relative to HDAC1 from two immunoblots; DUBs of interest and two non-targeting controls (siCON1 and siCON2) are indicated. Inset immunoblot panels show sections from the screen, highlighting DUB siRNAs that altered the HDAC expression ratio. The inset histogram shows an increase in HDAC1 and a decrease in HDAC2 expression for the siBAP1 samples from the replicate screens. **B.** BAP1 depletion decreases HDAC2 relative to HDAC1 in both cytosolic and nuclear protein extracts. A549 cells were transfected with 40 nM siRNAs for 72 hr. Quantification from immunoblotting shows the ratio of HDAC2 to HDAC1 expression from three independent experiments (error bars show SD, unpaired *t*-test, *P ≤ 0.05, **P ≤ 0.01). **C.** BAP1 depletion decreases HDAC2 and increases HDAC1 in other NSCLC cell lines. NCI-H460 or COR-L23 cells were transfected with 40 nM siRNAs for 72 hrs before immunoblotting of whole cell extracts. **D–E.** BAP1 siRNAs alter HDAC expression in MSTO-211H mesothelioma cells. Whole cell extracts were prepared 72 hr after transfection with siRNAs. A representative immunoblot **D.** and quantification from three independent experiments **E.** showing the HDAC2/HDAC1 ratio (left), and individual proteins normalized to actin (right); error bars show SD, one-way ANOVA with Tukey's post-hoc test: ****P ≤ 0.0001, ***P ≤ 0.001, **P ≤ 0.01, *P ≤ 0.05. **F.** Quantification of residual BAP1 for the siRNA experiments in B and D.

HDAC1 and HDAC2 are both subject to ubiquitin-mediated proteasomal degradation and several E3 ligases have been assigned [7–10]. However, ubiquitylation is a reversible process, and no DUBs have yet been described that may remove ubiquitin from HDAC1 or HDAC2. To identify DUBs that might be involved in specifically regulating the cellular abundance of either HDAC, we utilized a library of quality-controlled nucleoplasm lysates prepared from A549 NSCLC cells that had been depleted of each of the 92 human DUBs using a custom siRNA library, as described previously [14, 15]. We screened by immunoblotting and ranked the ratio of expression for HDAC2 relative to HDAC1 (Figure 2A). This ratio will reflect both the decrease in one HDAC (for example where a DUB is depleted that might normally rescue it from proteasomal degradation) and the compensatory increase for the opposite HDAC isoenzyme (as shown in Figure 1E). Whilst USP33 or USPL1 depletion increased the HDAC2/HDAC1 ratio, we identified USP27X and the tumor suppressor BAP1 as DUBs whose depletion lead to the most significant decrease in the HDAC2/HDAC1 ratio. In the case of BAP1, this indeed reflected both a decrease in HDAC2 and an increase in HDAC1 expression (Figure 2A).

This initial screen used pools of four siRNA sequences to target each DUB. To assess whether off-target effects might be responsible for altering HDAC abundance, we next transfected A549 cells with independent siRNA sequences. Statistically, it is unlikely that more than one siRNA would have the same off-target effect, so our criteria were that at least two individual siRNAs targeting a DUB produced a similar change in the HDAC2/HDAC1 ratio. This was not the case for USP33 or USPL1, and was marginal for USP27X (data not shown). In contrast, a reduction in HDAC2/HDAC1 was recapitulated by two siRNA sequences targeting BAP1 in A549 nucleoplasm extracts (Figure 2B). Both HDACs were also detected in cytosolic extracts, where their expression ratio was similarly affected by BAP1 siRNAs (Figure 2B), confirming that BAP1 depletion effects a change in abundance rather than sub-cellular relocalisation. BAP1 knockdown elicited this same switch in HDAC2/HDAC1 expression in two other NSCLC cell lines (Figure 2C), which endogenously express these HDACs at different levels (Figure 1B). As loss of BAP1 function is implicated in a high proportion of mesothelioma [16–19], we next asked whether we could recapitulate this effect in MSTO-211H cells, which were derived from a grade 4 biphasic mesothelioma and retain wild-type BAP1 expression [19]. Depletion of BAP1 consistently reduced HDAC2 and increased HDAC1 expression in whole cell protein extracts from these cells (Figure 2D & 2E). As before, siBAP1–3 more profoundly affected the HDAC2/HDAC1 expression ratio than siBAP1–5. Effects of BAP1 depletion were more marked in the MSTO-211H cells than in A549 cells, due to the relative knockdown efficiencies (Figure 2F).

**Figure 3:**
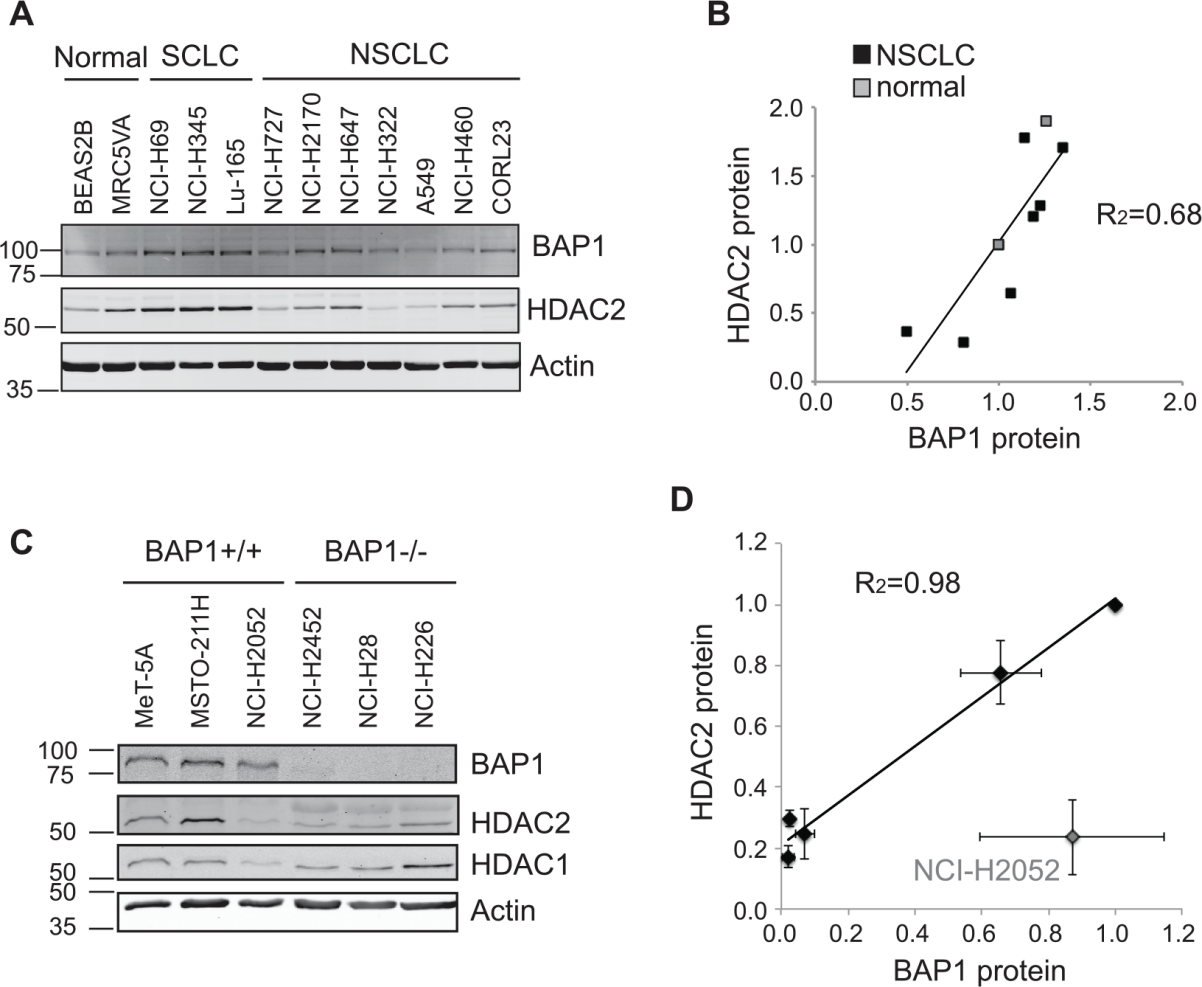
Endogenous BAP1 and HDAC2 expression are positively correlated **A–B.** Protein extracts from the panel of SCLC, NSCLC and normal lung derived cell lines shown in Figure 1B were analyzed for BAP1 expression. A representative immunoblot **A.** and quantification of relative protein expression **B.**, showing that HDAC2 protein levels correlate with those of BAP1 in non-neuroendocrine cells (linear regression R^2^ = 0.68). **C–D.** Immunoblotting in normal mesothelial cells and mesothelioma cell lines with differing genetic BAP1 status. A representative immunoblot **C.** and quantification of relative protein expression from three independent experiments **D.** show HDAC2 protein levels correlate with those of BAP1. NCI-H2052 cells are an outlier and have genetic loss of HDAC2 copy number (linear regression for other cell lines, R^2^ = 0.98).

In light of the effect of transient BAP1 depletion on HDAC expression, we hypothesized that variation in the endogenous expression of BAP1 in cancer cells may also influence HDAC levels. BAP1 is expressed to varying degrees across the panel of SCLC, NSCLC and normal lung cell lines, and is relatively under-expressed in the A549 cell line (Figure 3A) in which we had performed our initial screen (Figure 2A). In contrast, BAP1 is elevated in the SCLC cell lines, which express HDAC2 at high levels. Overall, we found a significant positive correlation between BAP1 and HDAC2 levels, as illustrated for the seven NSCLC and two normal lung cell lines in Figure 3B. In contrast there was no correlation between HDAC1 and BAP1 expression (linear regression R^2^ = 0.11).

As BAP1 depletion had a profound effect on the HDAC2/HDAC1 expression ratio in MSTO-211H cells (Figure 2D & E), we next examined the relationship between endogenous HDAC2 and BAP1 mutation status in a panel of established mesothelioma cell lines (Figure 3C). The MeT-5A cell line is derived from normal lung mesothelium, whilst the mesothelioma cell lines have differing BAP1 genetic status [19]: NCI-H2052 and MSTO-211H cells retain genetically wild-type BAP1, NCI-H28 and NCI-H226 cells are BAP1 null, and NCI-H2452 cells have an inactivating mutation in the BAP1 catalytic domain. Immunoblotting confirmed that BAP1 protein was expressed in the three wild-type lines, and was absent in the BAP1 null mesothelioma cell lines (Figure 3C). Very low levels of BAP1 were detected in NCI-H2452 cells, suggesting that BAP1 auto-deubiquitylation is not only required for nuclear localization [20] but perhaps also stability in these cells. Both HDAC1 and HDAC2 were expressed in the normal mesothelial MeT-5A cells, and HDAC1 was expressed at varying levels in the mesothelioma cell lines (Figure 3C). As predicted, HDAC2 expression was low in BAP1 null mesothelioma cell lines and overall showed a tight correlation with BAP1 expression status (Figure 3C & D). A notable exception was the NCI-H2052 cell line, which has loss of HDAC2 copy number (COSMIC, [21]) that likely accounts for low expression of HDAC2 even in the presence of BAP1.

**Figure 4:**
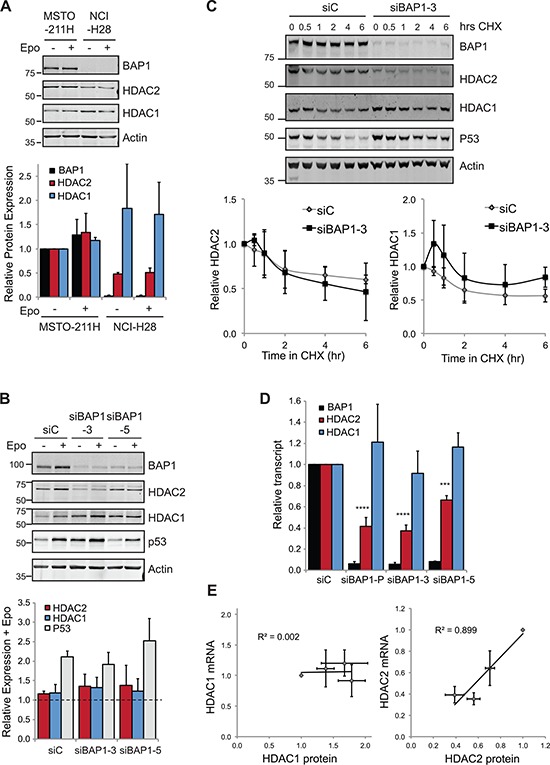
BAP1 regulates HDAC2 at the transcript level **A.** HDAC response to proteasome inhibition. Cells were treated with 50 nM epoxomicin (Epo) for 6 hr prior to whole cell lysis and immunoblotting. A representative immunoblot (top) and quantification of proteins from three independent experiments (below), error bars show SD. **B.** The effect of BAP1 depletion on HDAC2 levels is not rescued by proteasome inhibition. MSTO-211H cells were transfected with siRNAs for 72 hr and treated with 50 nM epoxomicin for the final 6 hr prior to whole cell lysis and immunoblotting. A representative immunoblot and quantification of proteins showing epoxomicin treated relative to untreated for each knockdown condition (three independent experiments, error bars show SD, no significant difference was determined between samples using one-way ANOVA with Dunnett's post-hoc test). **C.** BAP1 depletion does not significantly alter HDAC2 protein stability. MSTO-211H cells were transfected with siRNAs and treated with 10 μg/ml cycloheximide for the indicated times prior to whole cell protein extraction and immunoblotting. A representative immunoblot and quantification of HDACs (four independent experiments, error bars show SD, no significant difference was determined between samples by paired *t*-test). **D.** BAP1 depletion decreases HDAC2 mRNA. MSTO-211H cells were transfected with siRNAs as indicated and RNA extracted 72 hr later for qRT-PCR. Cq values for test genes were normalized to actin and expressed as 2^−[ΔΔCq]^ relative to the siC control (three independent experiments, error bars SD, one-way ANOVA with Dunnett's post-hoc test: ****P ≤ 0.0001, ***P ≤ 0.001). **E.** On depletion of BAP1, HDAC2 transcript is a good predictor of HDAC2 protein expression level.

This correlative expression further supported our functional data showing that BAP1 regulates HDAC2, so we investigated whether BAP1 might directly deubiquitylate and thus stabilize HDAC2. We first assessed whether inhibition of the proteasome led to accumulation of HDACs (Figure 4A). In BAP1 wild-type MSTO-211H cells we saw a marginal increase in HDAC2 levels in response to epoxomicin. Although we might expect a more profound response in BAP1-null cells, in fact we saw no increase in HDAC protein levels on epoxomicin treatment of NCI-H28, suggesting that turnover of HDAC2 is not a major limitation for its expression in this cell line. To determine whether HDAC2 turnover is BAP1-dependent in MSTO-211H cells, we compared rescue by proteasome inhibition in siRNA-transfected cells (Figure 4B). Expression of the labile protein P53 was clearly rescued by proteasome inhibition, and this was comparable between the knockdown conditions. As before, HDAC2 rescue by proteasome inhibition was modest, and was similar in BAP1-depleted or control cells. In addition, we found that BAP1 depletion did not reduce the stability of residual HDAC2 under conditions where translation was blocked (Figure 4C). Together these data suggest that BAP1 does not salvage HDAC2 from ubiquitin-mediated proteasomal degradation.

BAP1 is known to regulate H2A monoubiquitylation, as well as the polyubiquitylation of a number of transcription factors, so we wondered whether BAP1 might instead control transcription of HDAC2. To test this, we performed qRT-PCR in siRNA-transfected MSTO-211H cells and observed a marked decrease in HDAC2 transcript levels in cells depleted of BAP1 (Figure 4D). Indeed, we observed a strong correlation between HDAC2 protein (Figure 2E) and HDAC2 mRNA expression in these experiments (Figure 4E). In contrast, HDAC1 levels were not significantly affected by BAP1 depletion, and did not correlate with HDAC1 protein levels. We conclude that BAP1 primarily exerts its effects through regulating HDAC2 transcript abundance, which is coupled with compensation in HDAC2/HDAC1 protein expression.

**Figure 5:**
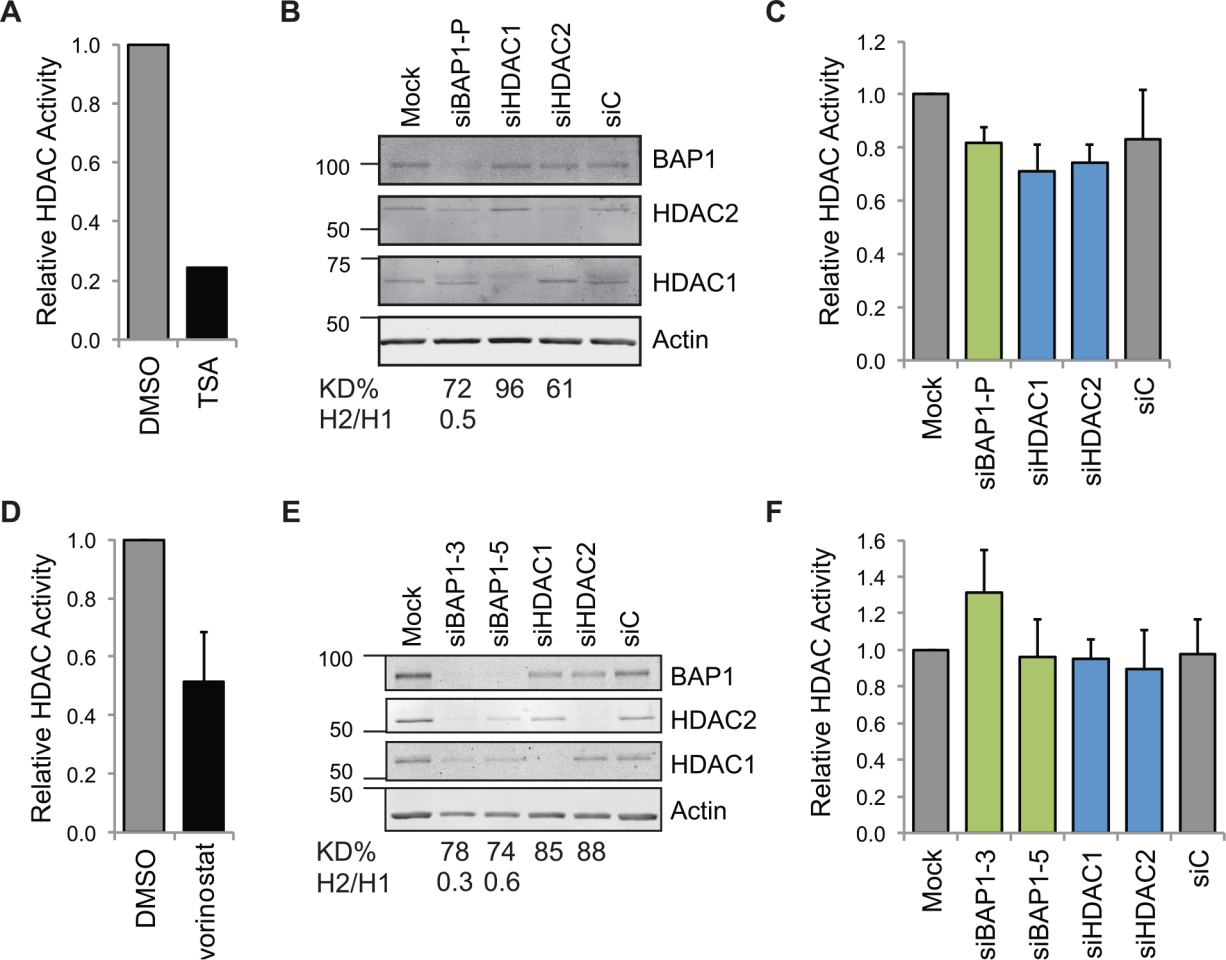
Neither HDAC nor BAP1 depletion alters total cellular HDAC activity HDAC activity was assessed in A549 cells **A–C.** and MSTO-211H cells **D–F.** As positive controls, cells were **A.** chronically treated with 250 nM of the pan-HDAC inhibitor TSA for 16 hr (mean of two independent experiments), or **D.** acutely treated with 5 μM vorinostat for 2 hr (mean of three independent experiments, error bar SD). BAP1 depletion does not affect activity towards the HDAC-GloI/II substrate. Cells were reverse transfected with 20 nM siRNA as indicated for 48 hr. Immunoblotting shows efficient depletion by reverse transfection of siRNA in the 96-well assay format, **B. E.,** the percentage efficiency for depletion of the target protein (KD%) and the ratio of HDAC2/HDAC1 expression (H2/H1) is indicated. For the HDAC-Glo assay run in parallel, **C. F.,** data show the mean of three **C.** or four **F.** independent experiments (error bars SD, no significant difference was determined between samples using one-way ANOVA with Dunnett's post-hoc test).

To establish whether the BAP1-dependent changes in HDAC1 and HDAC2 expression impact on overall cellular HDAC activity, we assayed the enzymatic activity of cell extracts towards an artificial HDAC-GloI/II substrate. In A549 cells, chronic treatment with the pan-HDAC inhibitor TSA abolished 75% of cellular HDAC activity as determined by this assay (Figure 5A). However, siRNA depletion of HDAC1, HDAC2 or BAP1 did not lead to significantly different cellular HDAC activity, suggesting that in each case the compensatory isoenzyme response maintains overall activity levels (Figure 5B and 5C). In MSTO-211H cells, acute treatment with vorinostat again reduced HDAC activity (Figure 5D), but we confirmed that depletion of either HDAC, or of BAP1, had no significant impact (Figure 5E and 5F).

**Figure 6:**
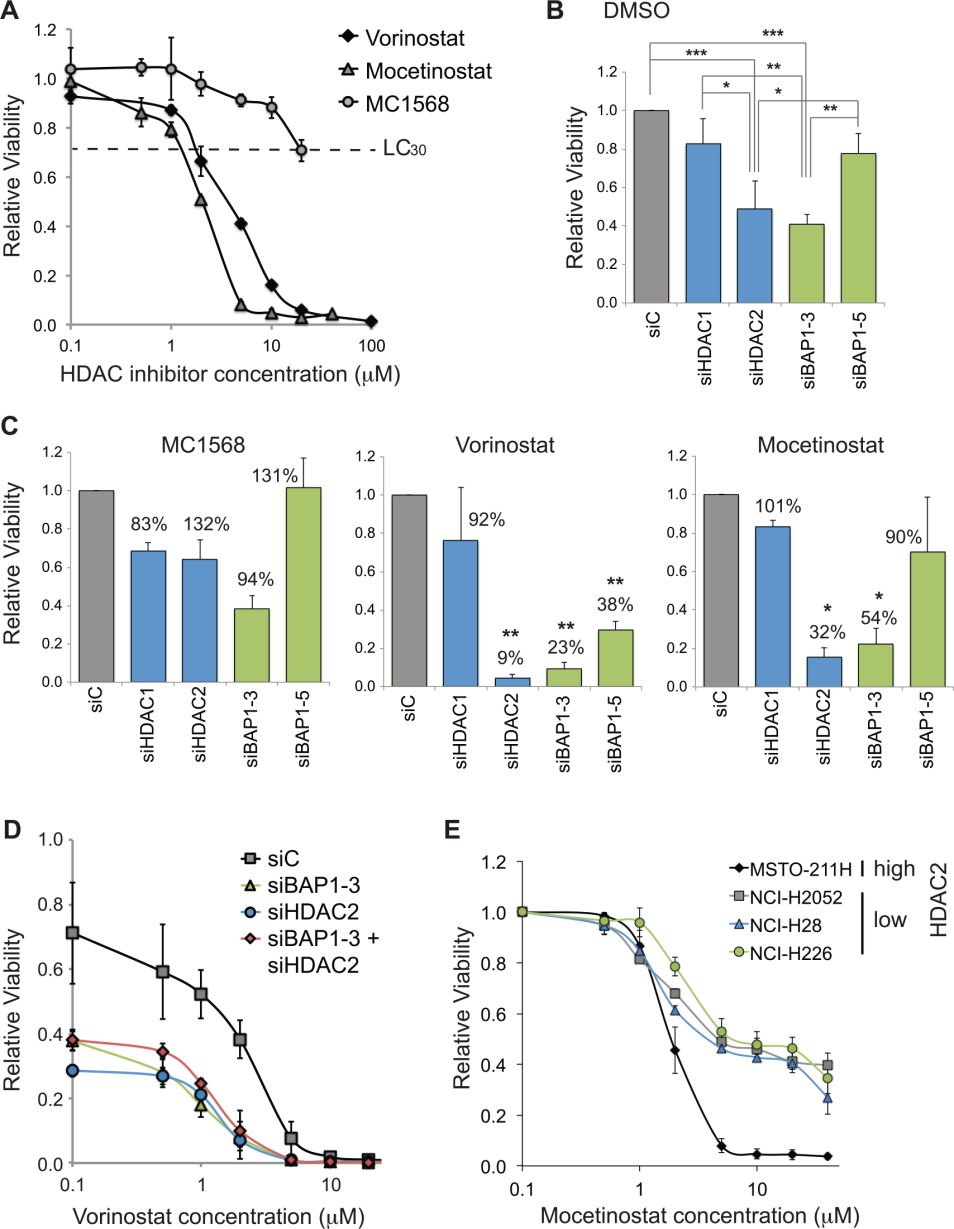
BAP1 status alters sensitivity of mesothelioma cells to HDAC inhibitors **A.** Dose response curves for HDAC inhibitors in MSTO-211H cells. Vorinostat (broad spectrum), mocetinostat (class I) and MC1568 (Class IIa) were added to cells at the indicated concentrations for 48 hr prior to CellTitre-Glo assay (error bars show SD of 4 technical replicates). MC1568 remains in aqueous solution at concentrations below 50 μM. **B–D.** HDAC2 or BAP1 depletion sensitizes cells to HDAC inhibitors. MSTO-211H cells were reverse transfected with 20 nM siRNA for 72 hr, and treated with vehicle (0.1% DMSO) **B.** or with HDAC inhibitors **C. D.** for the final 48 hr, before viable cell estimation by CellTitre-Glo assay (error bars show SD for three independent experiments). **B.** Treatment with vehicle: relative viability is plotted, compared to cells transfected with the siC control and treated with vehicle (one-way ANOVA with Tukey's post-hoc test: *P ≤ 0.05, **P ≤ 0.01, ***P ≤ 0.001). **C.** Treatment with the indicated HDAC inhibitors at their LC_30_: 20 μM MC1568, 2.5 μM vorinostat and 1.25 μM mocetinostat. The relative viability is plotted as a histogram for each drug treatment, this is based on comparison to cells transfected with siC and treated with that drug. For each bar on the histograms, percentage viability is also indicated, which compares the drug treated cells to the DMSO treated cells (shown in B) for the same knockdown condition; statistical significance for this comparison is shown (one-sample *t*-test, *P ≤ 0.05, **P ≤ 0.01). **D.** Dose response curves for vorinostat in HDAC2 and/or BAP1 depleted MSTO-211H cells; all data are normalized to siC transfected cells in 0.1% DMSO. **E.** Dose response curves for mocetinostat in a panel of mesothelioma cell lines with high (MSTO-211H) or low (other cell lines) constitutive HDAC2 expression. Data are normalized to 0.1 μM treatment for each cell line (error bars show SD for three independent experiments).

Although total HDAC activity was unchanged, we were interested to determine whether the BAP1-dependent switch in the HDAC2/HDAC1 isoenzymes affected cell viability when combined with HDAC inhibitors. We initially established the lethal concentration 30% for drugs that exhibit pan- or class-selective HDAC inhibition in MSTO-211H cells (Figure 6A). We next assessed the effects of BAP1 or HDAC siRNA on cell viability. While HDAC1 depletion had little effect, depletion of HDAC2 significantly reduced cell viability, suggesting that MSTO-211H cells are dependent on HDAC2 for survival (Figure 6B, DMSO). Importantly, BAP1 depletion also reduced viability. This effect was more pronounced for siBAP1–3 than for siBAP1–5, mirroring their respective effects on HDAC2 expression (Figure 2).

We then explored whether MSTO-211H cells depleted of these proteins would be more sensitive to HDAC inhibitors applied at their LC_30_ (Figure 6C). None of the siRNA transfected cells were significantly sensitized to the class IIa selective inhibitor MC1568. Neither did addition of pan-HDAC (vorinostat) or of class I selective (mocetinostat) inhibitors significantly influence the survival of HDAC1 depleted cells. In contrast, HDAC2 depleted cells were sensitized to both HDAC inhibitors, with significantly decreased viability compared to DMSO alone. Importantly, BAP1 knockdown also increased sensitivity to HDAC inhibition in a similar fashion, decreasing cell survival with both vorinostat and mocetinostat.

In dose response curves, the LC_50_ of vorinostat halves from 2.6 μM in siC-treated cells to 1.3 μM with HDAC2 siRNA and 1.2 μM in BAP1 depleted cells. Crucially, concomitant BAP1 and HDAC2 depletion did not additively reduce cell viability in the presence of vorinostat (Figure 6D). This is consistent with loss of BAP1 mediating increased drug sensitivity through its ability to lower HDAC2 levels. Lastly, we assessed the relative HDAC inhibitor sensitivity of three established mesothelioma cells with constitutively low HDAC2 expression together with the MSTO-211H cells that retain high HDAC2 expression (Figure 3C). Intriguingly, in contrast to our data for transient HDAC2 knockdown, the constitutively HDAC2-deficient mesothelioma cell lines were more resistant to mocetinostat (Figure 6E). We found that the LC_50_ of the HDAC2-deficient lines NCI-H2052, NCI-H28 and NCI-H226 was 4.0, 4.5 and 4.9 μM, respectively, compared to 2.4 μM for MSTO-211H cells. Furthermore, in the HDAC2-deficient lines, around a third of cells remained viable at much higher doses, suggesting they have adapted to become less dependent on HDAC2 for viability.

In conclusion, loss of BAP1 in cell line models of thoracic malignancies alters expression of HDAC2. This in turn modulates the sensitivity of these cells to clinically relevant HDAC inhibitors.

## DISCUSSION

We report here an unbiased DUB siRNA screen that has identified BAP1 as a regulator of HDAC2 expression in lung cancer and mesothelioma cells. BAP1 was first identified as tumor suppressor, when mutations were reported in lung cancer cell lines [22]. In fact, somatic BAP1 mutation occurs in only around 1% of lung adenocarcinoma [23, 24], but is far more prevalent in uveal melanoma [25, 26], mesothelioma [18, 19] and renal clear cell carcinoma [27]. Germline BAP1 mutation underpins a cancer predisposition syndrome [18, 25, 26, 28–30] and BAP1 protein expression is reportedly lost in around 50% of NSCLC, colon carcinoma, uveal melanoma and kidney cancers [31–37]. Mechanistically, BAP1 removes monoubiquitin from histone H2A [38], or stabilizes proteins like BRCA1 and HCFC1 by removing polyubiquitin chains [22, 39, 40], whilst many transcription factors and chromatin modifiers have been identified as BAP1 interacting proteins [41, 42].

Here we show for the first time that BAP1 modulates expression of another chromatin-associated protein, HDAC2. However, this is mediated not through direct deubiquitylation of HDAC2, but through regulating HDAC2 transcript abundance. Loss of BAP1 increases H2A monoubiquitylation, which is transcriptionally repressive, suggesting one route by which this may be mediated. However, indirect effects of BAP1 via transcription factors that control HDAC2 transcription, or through alteration of HDAC2 transcript stability, might also play roles. We find that BAP1 loss promotes a switch from HDAC2 to HDAC1. This arises due to a well-established reciprocal compensatory mechanism, so that when HDAC2 levels drop, HDAC1 levels rise. In cell lines and in transgenic mice, this crosstalk between HDAC1 and HDAC2 is not mediated by altered transcription, but through changes in either protein synthesis or turnover [43, 44].

It is interesting that our current screening strategy did not uncover any DUBs that reverse HDAC2 polyubiquitylation and so stabilize the HDAC2 protein. In fact, our data also suggest that HDAC2 turnover by proteasomal degradation may not be a major determinant of its abundance under normal growth conditions in these cell lines. Strategies to stimulate HDAC2 turnover, such as the response to cigarette smoke [5], may aid future screens for DUBs that directly antagonize ubiquitylation of HDAC2.

When we compared HDAC expression in a panel of cell lines, we found that HDAC2 transcript abundance was not a good predictor of HDAC2 protein level (Figure 1D). This is not unusual, in fact global studies report that the major determinant of protein abundance is neither transcription nor protein degradation, but is instead translation [45]. Intriguingly though, when we deplete BAP1 leading to a reduction in HDAC2 mRNA, this transcript now becomes a good predictor of HDAC2 protein expression (Figure 4E). Taken together, these data suggest that although HDAC1 and HDAC2 are normally co-transcribed, the HDAC2 transcript is in excess of the cellular requirements for HDAC2 protein. However, in the absence of BAP1 their transcription is disengaged and availability of HDAC2 mRNA may now limit HDAC2 protein expression.

The clinical relevance of BAP1 loss-of-function in mesothelioma remains under debate, with conflicting reports that it may be preferentially associated with epithelioid morphology, later age of presentation, or overall survival [16, 19, 36, 46] and there are as yet no studies that provide a rationale for specific targeted therapies. Up to 80% of mesothelioma are directly attributable to asbestos exposure [47] and it was recently shown that mice with heterozygous germline BAP1 knockout were predisposed to develop mesothelioma upon asbestos exposure [48]. Interestingly, somatic BAP1 mutation is reported to be more common in current or ex-smokers who develop mesothelioma [46] and could conceivably compound HDAC2 instability in response to cigarette smoke [5].

BAP1 loss might also be associated with cancer progression and metastasis. Clinically BAP1 mutation was initially linked to more aggressive, metastasizing uveal melanoma [26], whilst subsequent cell models suggested that BAP1 loss induces a stem cell-like phenotype [49]. In mesothelioma, BAP1 loss is more common in a clinical sub-group that exhibit less evidence of EMT [50], but on asbestos exposure BAP1^+/–^ mice develop more aggressive tumors, that invade other organs, than their wild-type littermates [48]. In the absence of a carcinogenic insult however, BAP1 loss may not promote cell growth. Indeed uveal melanoma cells stably expressing BAP1 shRNA show no difference in their *in vitro* or *in vivo* growth kinetics [49] and BAP1^+/–^ mice do not spontaneously develop tumors [48]. In several studies, BAP1 loss is in fact reported to slow cell growth through a delayed but more permissive G1/S cell cycle checkpoint [19, 40]. Indeed, on transient BAP1 depletion in MSTO-211H cells, we observed an approximate 50% reduction in viable cell number, comparable with previous data for this cell line [19].

These data suggest cytotoxic drugs targeting rapidly dividing cells are not apposite for BAP-deficient mesothelioma. Indeed, mesothelioma shows low chemo- and radio-sensitivity and targeted therapies are urgently required [51]. Synthetic lethal approaches offer new opportunities to exploit essential survival pathways in cancer cells with BAP1 loss-of-function. Several cancer drugs have been identified that exhibit synthetic lethality with specific tumor suppressors, most notably BRCA1 mutation/PARP inhibitors [52]. As BAP1 interacts with BRCA1, PARP inhibitors might also exhibit synthetic lethality with BAP1 mutation. However, whilst this was seen in BAP1 knockout chicken lymphoma cells [53], no synthetic effects were found in human mesothelioma cells [19] emphasizing the importance of cellular context. Our initial data suggest a potential relationship between BAP1 and HDAC inhibitors that could be exploited instead.

Despite early promise, histone deacetylase inhibitors have failed to show significant clinical activity in solid tumors. In mesothelioma for example, while an early phase trial found some evidence for activity of vorinostat, a subsequent large phase III study did not show any benefit for vorinostat in second line treatment [54, 55]. The failure of this trial understandably led to a dampening of enthusiasm for HDAC inhibitors in mesothelioma. It is however feasible that the lack of benefit in the overall trial could mask a subgroup of patients who derived benefit from treatment. The need to stratify NSCLC patients based on EGFR mutation for EGFR inhibitor clinical trials [56–58] highlights the importance of identifying biomarkers that can predict for response.

We found that despite the effect on HDAC2 expression, loss of BAP1 does not impact on total cellular HDAC activity, suggesting the compensatory increase in HDAC1 maintains cellular HDAC activity. This is consistent with other reports of the HDAC relationship, for example in mice with targeted inactivation of either HDAC1 or HDAC2 in the epidermis, there is reciprocal upregulation of the opposite isoenzyme with no reduction in total HDAC activity [43]. Specific roles for the class I HDAC isoenzymes are however increasingly recognized, and this BAP1-dependent switch in the prevalence of HDAC1 and HDAC2 may have functional implications. Indeed we find that HDAC2 appears more important than HDAC1 in maintaining viability of the BAP1 positive mesothelioma cell line MSTO-211H. Of particular importance in a clinical setting, this could impact on the sensitivity of cancer cells to HDAC inhibitors. Despite the maintenance of total HDAC activity, we found increased sensitivity to HDAC inhibitors on depletion of HDAC2 or BAP1, but not of HDAC1 (Figure 6). This lends support to HDAC isoenzyme-specific roles, and suggests HDAC2 specific inhibitors might enable more precise targeted therapy.

The relationship between BAP1 expression and HDAC inhibitor sensitivity has not previously been explored in mesothelioma. In uveal melanoma cell lines, HDAC inhibitors partially rescue the loss of melanocytic differentiation associated with BAP1 depletion [49, 59]. Furthermore, stable BAP1 depletion in a uveal melanoma cell line leads to sensitization to the HDAC inhibitor valproic acid and decreased viability. Although changes in H2A ubiquitylation status were observed in this experiment [59], the mechanism for HDAC inhibitor sensitization remains unclear. Our data, which show HDAC2 expression to be contingent on BAP1, provide the first mechanism to underpin differential sensitivity of BAP1-deficient cells to HDAC inhibitors.

Significantly however, our data show that transient loss of BAP1 expression is not equivalent to its genetic inactivation in cell lines that have been in long term culture, raising a number of points. Firstly, siRNA screening has allowed us to identify a new target of the deubiquitylase BAP1; however inferring clinical biomarkers from transient depletion of BAP1 might give deceptive results. Indeed, our data suggest that cells adapt to genetic BAP1 loss, such that sensitivity to HDAC inhibitors is reduced. While our findings will need to be confirmed in other models, this is potentially of clinical significance, as HDAC inhibitors are currently being investigated in clinical trials for uveal melanoma in which BAP1 mutations are common. On the other hand, our data suggest that cancer cells develop compensatory mechanisms to survive without BAP1; identifying these mechanisms may provide new opportunities for synthetic lethal strategies. Well-defined models of genetic BAP1 loss are now needed to allow screening for biomarkers and new targets. These, alongside clinical studies to assess the prevalence of BAP1-dependent HDAC2 loss, will help establish how BAP1 status affects pan or class-selective HDAC inhibitor sensitivity.

## MATERIALS AND METHODS

### Cell culture

A549 cells (obtained from ECACC) were cultured in Dulbecco's modified Eagle's medium (DMEM) supplemented with 10% fetal bovine serum (FBS) and 1% non-essential amino acids, at 37°C and 5% CO_2_. The mesothelioma cell lines MSTO-211H, NCI-H2052, NCI-H28, NCI-H226 and NCI-H2452 (from ATCC) and all other lung cancer cell lines (sourced as previously described [60]) were cultured in RPMI supplemented with 10% FBS. The transformed normal mesothelial cell line MeT-5A (ATCC) was cultured in Medium 199 with added 10% FBS, 2% HEPES, 0.1% Trace Elements B, 0.02% (100 μg/ml) EGF, 0.028% (1 mg/ml) Hydrocortisone, 0.05% (10 mg/ml) Insulin, and 0.01% (2 mg/ml) selenium acid.

### RNA and protein extraction

To screen for correlative expression, total RNA and protein were extracted in parallel from the same samples using an RNA/DNA/protein purification kit (Norgen). In other experiments, total RNA was extracted using RNeasy kits (Qiagen) with on-column DNase digestion. Fractionated protein extracts were prepared by sequential extraction in NP-40 buffer (0.5% NP-40, 25 mM Tris pH7.9, 150 mM NaCl), Dignam buffer C (20 mM HEPES pH7.9, 25% glycerol, 0.42 mM NaCl, 1.5 mM MgCl, 0.2 mM EDTA) and Laemmli buffer (50 mM Tris pH6.8, 2% SDS, 10% glycerol). Alternatively, whole cell protein extracts were prepared by direct addition to cells of hot Laemmli buffer and incubation at 110°C for 10mins with intermittent vortexing. The protein concentration of each sample was determined after suitable dilution using a Bicinchoninic Acid (BCA) assay (Thermo Scientific).

### Quantitative RT-PCR

cDNA was reverse transcribed from 1 μg RNA with RevertAid H-minus M-MuLV reverse transcriptase (Fermentas) using an oligodT primer (Promega). Quantitative real-time RT-PCR (qRT-PCR) was performed in triplicate using SYBR Green Supermix and a real-time PCR detection system (Bio-rad). The primer pairs used were: BAP1 (For: 5′-CAGCCCTGAGAGCAAAGGATATG-3′, Rev: 5′-ATGGTCCGCACTGCACTAAG-3′), HDAC1 (For: 5′-ACGGGATTGATGACGAGTCCTATG-3′, Rev: 5′-TGAGCCACACTGTAAGACCACC-3′), HDAC2 (For: 5′-TTACTGATGCTTGGAGGAGGTGGC-3′, Rev: 5′- TGG ACACCAGGTGCATGAGGTAAC-3′) and β-actin (ACTB, For: 5′-CACCTTCTACAATGAGCTGCGTGTG-3′, Rev: 5′-ATAGCACAGCCTGGATAGCAACGTAC-3′). Samples underwent 2-step amplification at 94°C (30 s) and 60°C (60 s); melt curves were analyzed after 40 cycles. The Cq values for test genes were normalized to ACTB and relative expression represented as 2^−[ΔΔCq]^.

### Immunoblotting and antibodies

Equal amounts of lysates for comparator samples were subject to immunoblotting. Following resolution by 10% SDS-PAGE, proteins were transferred to BiotraceNT membrane (VWR) and incubated with primary antibodies. Antibodies used were: mouse anti-BAP1 (C-4, Santa Cruz), anti-P53 (sc-126, Santa Cruz) and anti-β-actin (ab6276, Abcam), rabbit anti-HDAC2 (H54, Santa Cruz), goat anti-HDAC1 (C19, Santa Cruz). Proteins were visualized using donkey anti-mouse, anti-rabbit or anti-sheep secondary antibodies conjugated to the IRDyes IR680-LT, or IR800 (LI-COR) and the LI-COR Odyssey 2.1 system; 16-bit images were analyzed and quantified using the Odyssey analysis software.

### RNA interference and DUB siRNA library screen

A custom designed DUB siRNA library consisting of pools of four oligos for each of 92 human DUBs (Qiagen) and control siRNAs were previously used to prepare a library of fractionated protein extracts from A549 cells [14]. To screen for the effect of each DUB on HDAC levels, samples from this nuclear extract library were divided across four 4%–12% gradient gels that were run and processed in parallel. Following immunoblotting, the HDACs were imaged in separate channels and bands were quantified using the Odyssey system; the amount of HDAC2 was expressed relative to the amount of HDAC1. The value for each sample was then normalized to the median value for the corresponding immunoblot. Mean values were derived from two independent immunoblotting runs, before collation and ranking of the dataset. The individual siRNA sequences from each pool were used for initial validation. Two of these siRNAs hs_BAP1_3 (SI00066710) and hs_BAP1_5 (SI03036390) were used for follow-up studies together with hs_HDAC1_6 (SI02663472) and hs_HDAC2_1 (SI00434952) (Qiagen). Non-targeting control siRNAs used were siC (AllStars, Qiagen), and siCON1 (D-001210–01) or siCON2 (D-001210–02) from Dharmacon. For immunoblotting experiments, cells were seeded at 6×10^4^ (A549 and NCI-H460) or 1.5×10^5^ (MSTO-211H and COR-L23) cells per well in 6-well plates, respectively, transfected the following day with 40 nM siRNA using Oligofectamine (Invitrogen), and harvested after 72 hr.

### Inhibitors

Cells were treated with the following HDAC inhibitors: pan-HDAC vorinostat (Selleck) and trichostatin A (TSA, Sigma), class I selective mocetinostat (MGCD01032, Selleck) and class IIa selective MC1568 (Selleck). In cycloheximide chase experiments, cycloheximide (Sigma) was added to cells at 10 μg/ml for periods of up to 6 hr. In proteasome inhibition experiments, epoxomicin (Calbiochem) was added to cells at 50 nM for 6 hr.

### HDAC activity assay

A549 or MSTO-211H cells were seeded in 96-well plates at a density of 4000 or 6000 cells per well, respectively. Treatment with 250 nM TSA for 16 hr, or 5 μM vorinostat for 2 hr, was used a positive control in siRNA experiments. Cells were reverse transfected using Lipofectamine RNAiMax (Invitrogen) with 20 nM siRNAs targeting HDACs or DUBs, or a non-targeting siRNA (siC) or reagent only (mock). Total cellular HDAC activity was assayed 48 hr later using the HDAC GloI/II Assay (Promega) in non-lytic format and normalized for cell viability. Parallel lysates were collected from 96-well plates for immunoblotting; equivalent volumes were loaded without BCA assay.

### Cell viability assays

The cellular toxicity of HDAC inhibitors was titrated in untransfected mesothelioma cell lines. For knockdown experiments, 4000 MSTO-211H cells were seeded into 96-well plates and reverse transfected for 72 hr with 20 nM siRNA using RNAiMax. Cells were treated with vehicle (0.1% DMSO) or HDAC inhibitors at their LC_30_ (2.5 μM vorinostat, 1.25 μM mocetinostat, 20 μM MC1568) for the final 48 hr prior to analysis, with the media and drug replaced every 24 hr. The relative number of viable cells were determined, based on proportionality with the amount of ATP present, using the CellTitre-Glo assay (Promega).

### Statistics

All statistical tests were performed using GraphPad Prism version 6.00 for Mac.

## References

[1] Choudhary C, Kumar C, Gnad F, Nielsen ML, Rehman M, Walther TC, Olsen JV, Mann M (2009). Lysine acetylation targets protein complexes and co-regulates major cellular functions. Science.

[2] Lane AA, Chabner BA (2009). Histone deacetylase inhibitors in cancer therapy. J Clin Oncol.

[3] Moser MA, Hagelkruys A, Seiser C (2014). Transcription and beyond: the role of mammalian class I lysine deacetylases. Chromosoma.

[4] Senese S, Zaragoza K, Minardi S, Muradore I, Ronzoni S, Passafaro A, Bernard L, Draetta GF, Alcalay M, Seiser C, Chiocca S (2007). Role for histone deacetylase 1 in human tumor cell proliferation. Mol Cell Biol.

[5] Adenuga D, Yao H, March TH, Seagrave J, Rahman I (2009). Histone deacetylase 2 is phosphorylated, ubiquitinated, and degraded by cigarette smoke. Am J Respir Cell Mol Biol.

[6] Segre CV, Chiocca S (2011). Regulating the regulators: the post-translational code of class I HDAC1 and HDAC2. J Biomed Biotechnol.

[7] Gaughan L, Logan IR, Neal DE, Robson CN (2005). Regulation of androgen receptor and histone deacetylase 1 by Mdm2-mediated ubiquitylation. Nucleic Acids Res.

[8] Oh YM, Kwon YE, Kim JM, Bae SJ, Lee BK, Yoo SJ, Chung CH, Deshaies RJ, Seol JH (2009). Chfr is linked to tumour metastasis through the downregulation of HDAC1. Nat Cell Biol.

[9] Kramer OH, Zhu P, Ostendorff HP, Golebiewski M, Tiefenbach J, Peters MA, Brill B, Groner B, Bach I, Heinzel T, Gottlicher M (2003). The histone deacetylase inhibitor valproic acid selectively induces proteasomal degradation of HDAC2. EMBO J.

[10] Zhang J, Kan S, Huang B, Hao Z, Mak TW, Zhong Q (2011). Mule determines the apoptotic response to HDAC inhibitors by targeted ubiquitination and destruction of HDAC2. Genes Dev.

[11] Marks PA (2007). Discovery and development of SAHA as an anticancer agent. Oncogene.

[12] Grassadonia A, Cioffi P, Simiele F, Iezzi L, Zilli M, Natoli C (2013). Role of Hydroxamate-Based Histone Deacetylase Inhibitors (Hb-HDACIs) in the Treatment of Solid Malignancies. Cancers.

[13] Ononye SN, van Heyst M, Falcone EM, Anderson AC, Wright DL (2012). Toward isozyme-selective inhibitors of histone deacetylase as therapeutic agents for the treatment of cancer. Pharmaceutical patent analyst.

[14] Sacco JJ, Yau TY, Darling S, Patel V, Liu H, Urbe S, Clague MJ, Coulson JM (2014). The deubiquitylase Ataxin-3 restricts PTEN transcription in lung cancer cells. Oncogene.

[15] Faronato M, Patel V, Darling S, Dearden L, Clague MJ, Urbe S, Coulson JM (2013). The deubiquitylase USP15 stabilizes newly synthesized REST and rescues its expression at mitotic exit. Cell Cycle.

[16] Yoshikawa Y, Sato A, Tsujimura T, Emi M, Morinaga T, Fukuoka K, Yamada S, Murakami A, Kondo N, Matsumoto S, Okumura Y, Tanaka F, Hasegawa S (2012). Frequent inactivation of the BAP1 gene in epithelioid-type malignant mesothelioma. Cancer Sci.

[17] Carbone M, Korb Ferris L, Baumann F, Napolitano A, Lum CA, Flores EG, Gaudino G, Powers A, Bryant-Greenwood P, Krausz T, Hyjek E, Tate R, Friedberg J (2012). BAP1 cancer syndrome: malignant mesothelioma, uveal and cutaneous melanoma, and MBAITs. J Transl Med.

[18] Testa JR, Cheung M, Pei J, Below JE, Tan Y, Sementino E, Cox NJ, Dogan AU, Pass HI, Trusa S, Hesdorffer M, Nasu M, Powers A (2011). Germline BAP1 mutations predispose to malignant mesothelioma. Nat Genet.

[19] Bott M, Brevet M, Taylor BS, Shimizu S, Ito T, Wang L, Creaney J, Lake RA, Zakowski MF, Reva B, Sander C, Delsite R, Powell S (2011). The nuclear deubiquitinase BAP1 is commonly inactivated by somatic mutations and 3p21.1 losses in malignant pleural mesothelioma. Nat Genet.

[20] Mashtalir N, Daou S, Barbour H, Sen NN, Gagnon J, Hammond-Martel I, Dar HH, Therrien M (2014). Autodeubiquitination protects the tumor suppressor BAP1 from cytoplasmic sequestration mediated by the atypical ubiquitin ligase UBE2O. Mol Cell.

[21] Forbes SA, Bindal N, Bamford S, Cole C, Kok CY, Beare D, Jia M, Shepherd R, Leung K, Menzies A, Teague JW, Campbell PJ, Stratton MR (2011). COSMIC: mining complete cancer genomes in the Catalogue of Somatic Mutations in Cancer. Nucleic Acids Res.

[22] Jensen DE, Proctor M, Marquis ST, Gardner HP, Ha SI, Chodosh LA, Ishov AM, Tommerup N, Vissing H, Sekido Y, Minna J, Borodovsky A, Schultz DC (1998). BAP1: a novel ubiquitin hydrolase which binds to the BRCA1 RING finger and enhances BRCA1-mediated cell growth suppression. Oncogene.

[23] Ding L, Getz G, Wheeler DA, Mardis ER, McLellan MD, Cibulskis K, Sougnez C, Greulich H, Muzny DM, Morgan MB, Fulton L, Fulton RS, Zhang Q (2008). Somatic mutations affect key pathways in lung adenocarcinoma. Nature.

[24] Goldstein AM (2011). Germline BAP1 mutations and tumor susceptibility. Nat Genet.

[25] Wiesner T, Obenauf AC, Murali R, Fried I, Griewank KG, Ulz P, Windpassinger C, Wackernagel W, Loy S, Wolf I, Viale A, Lash AE, Pirun M (2011). Germline mutations in BAP1 predispose to melanocytic tumors. Nat Genet.

[26] Harbour JW, Onken MD, Roberson ED, Duan S, Cao L, Worley LA, Council ML, Matatall KA, Helms C, Bowcock AM (2010). Frequent mutation of BAP1 in metastasizing uveal melanomas. Science.

[27] Guo G, Gui Y, Gao S, Tang A, Hu X, Huang Y, Jia W, Li Z, He M, Sun L, Song P, Sun X, Zhao X (2011). Frequent mutations of genes encoding ubiquitin-mediated proteolysis pathway components in clear cell renal cell carcinoma. Nat Genet.

[28] Abdel-Rahman MH, Pilarski R, Cebulla CM, Massengill JB, Christopher BN, Boru G, Hovland P, Davidorf FH (2011). Germline BAP1 mutation predisposes to uveal melanoma, lung adenocarcinoma, meningioma, and other cancers. J Med Genet.

[29] Popova T, Hebert L, Jacquemin V, Gad S, Caux-Moncoutier V, Dubois-d'Enghien C, Richaudeau B, Renaudin X, Sellers J, Nicolas A, Sastre-Garau X, Desjardins L, Gyapay G (2013). Germline BAP1 mutations predispose to renal cell carcinomas. Am J Hum Genet.

[30] Murali R, Wiesner T, Scolyer RA (2013). Tumours associated with BAP1 mutations. Pathology.

[31] Fan LH, Tang LN, Yue L, Yang Y, Gao ZL, Shen Z (2012). BAP1 is a good prognostic factor in advanced non-small cell lung cancer. Clin Invest Med.

[32] Tang J, Xi S, Wang G, Wang B, Yan S, Wu Y, Sang Y, Wu W, Zhang R, Kang T (2013). Prognostic significance of BRCA1-associated protein 1 in colorectal cancer. Med Oncol.

[33] Shah AA, Bourne TD, Murali R (2013). BAP1 protein loss by immunohistochemistry: a potentially useful tool for prognostic prediction in patients with uveal melanoma. Pathology.

[34] Kapur P, Christie A, Raman JD, Then MT, Nuhn P, Buchner A, Bastian P, Seitz C, Shariat SF, Bensalah K, Rioux-Leclercq N, Xie XJ, Lotan Y (2014). BAP1 Immunohistochemistry Predicts Outcomes in a Multi-Institutional Cohort with Clear Cell Renal Cell Carcinoma. J Urol.

[35] Joseph RW, Kapur P, Serie DJ, Eckel-Passow JE, Parasramka M, Ho T, Cheville JC, Frenkel E, Rakheja D, Brugarolas J, Parker A (2014). Loss of BAP1 protein expression is an independent marker of poor prognosis in patients with low-risk clear cell renal cell carcinoma. Cancer.

[36] Arzt L, Quehenberger F, Halbwedl I, Mairinger T, Popper HH (2014). BAP1 protein is a progression factor in malignant pleural mesothelioma. Pathol Oncol Res.

[37] Kalirai H, Dodson A, Faqir S, Damato BE, Coupland SE (2014). Lack of BAP1 protein expression in uveal melanoma is associated with increased metastatic risk and has utility in routine prognostic testing. Br J Cancer.

[38] Scheuermann JC, de Ayala Alonso AG, Oktaba K, Ly-Hartig N, McGinty RK, Fraterman S, Wilm M, Muir TW, Muller J (2010). Histone H2A deubiquitinase activity of the Polycomb repressive complex PR-DUB. Nature.

[39] Misaghi S, Ottosen S, Izrael-Tomasevic A, Arnott D, Lamkanfi M, Lee J, Liu J, O'Rourke K, Dixit VM, Wilson AC (2009). Association of C-terminal ubiquitin hydrolase BRCA1-associated protein 1 with cell cycle regulator host cell factor 1. Mol Cell Biol.

[40] Machida YJ, Machida Y, Vashisht AA, Wohlschlegel JA, Dutta A (2009). The deubiquitinating enzyme BAP1 regulates cell growth via interaction with HCF-1. J Biol Chem.

[41] Sowa ME, Bennett EJ, Gygi SP, Harper JW (2009). Defining the human deubiquitinating enzyme interaction landscape. Cell.

[42] Dey A, Seshasayee D, Noubade R, French DM, Liu J, Chaurushiya MS, Kirkpatrick DS, Pham VC, Lill JR, Bakalarski CE, Wu J, Phu L, Katavolos P (2012). Loss of the tumor suppressor BAP1 causes myeloid transformation. Science.

[43] Winter M, Moser MA, Meunier D, Fischer C, Machat G, Mattes K, Lichtenberger BM, BrunMeir R, Weissmann S, Murko C, Humer C, Meischel T, Brosch G (2013). Divergent roles of HDAC1 and HDAC2 in the regulation of epidermal development and tumorigenesis. EMBO J.

[44] Jurkin J, Zupkovitz G, Lagger S, Grausenburger R, Hagelkruys A, Kenner L, Seiser C (2011). Distinct and redundant functions of histone deacetylases HDAC1 and HDAC2 in proliferation and tumorigenesis. Cell Cycle.

[45] Schwanhausser B, Busse D, Li N, Dittmar G, Schuchhardt J, Wolf J, Chen W, Selbach M (2011). Global quantification of mammalian gene expression control. Nature.

[46] Zauderer MG, Bott M, McMillan R, Sima CS, Rusch V, Krug LM, Ladanyi M (2013). Clinical characteristics of patients with malignant pleural mesothelioma harboring somatic BAP1 mutations. J Thorac Oncol..

[47] Carbone M, Kratzke RA, Testa JR (2002). The pathogenesis of mesothelioma. Semin Oncol.

[48] Xu J, Kadariya Y, Cheung M, Pei J, Talarchek J, Sementino E, Tan Y, Menges CW, Cai KQ, Litwin S, Peng H, Karar J, Rauscher FJ (2014). Germline mutation of Bap1 accelerates development of asbestos-induced malignant mesothelioma. Cancer Res.

[49] Matatall KA, Agapova OA, Onken MD, Worley LA, Bowcock AM, Harbour JW (2013). BAP1 deficiency causes loss of melanocytic cell identity in uveal melanoma. BMC Cancer.

[50] de Reynies A, Jaurand MC, Renier A, Couchy G, Hysi I, Elarouci N, Galateau-Salle F, Copin MC, Hofman P, Cazes A, Andujar P, Imbeaud S, Petel F (2014). Molecular classification of malignant pleural mesothelioma: identification of a poor prognosis subgroup linked to the epithelial-to-mesenchymal transition. Clin Cancer Res.

[51] Zucali PA, De Vincenzo F, Simonelli M, Santoro A (2009). Future developments in the management of malignant pleural mesothelioma. Expert Rev Anticancer Ther.

[52] Farmer H, McCabe N, Lord CJ, Tutt AN, Johnson DA, Richardson TB, Santarosa M, Dillon KJ, Hickson I, Knights C, Martin NM, Jackson SP, Smith GC (2005). Targeting the DNA repair defect in BRCA mutant cells as a therapeutic strategy. Nature.

[53] Yu H, Pak H, Hammond-Martel I, Ghram M, Rodrigue A, Daou S, Barbour H, Corbeil L, Hebert J, Drobetsky E, Masson JY, Di Noia JM (2014). Tumor suppressor and deubiquitinase BAP1 promotes DNA double-strand break repair. Proc Natl Acad Sci U S A.

[54] Kelly WK, O'Connor OA, Krug LM, Chiao JH, Heaney M, Curley T, MacGregore-Cortelli B, Tong W, Secrist JP, Schwartz L, Richardson S, Chu E, Olgac S (2005). Phase I study of an oral histone deacetylase inhibitor, suberoylanilide hydroxamic acid, in patients with advanced cancer. J Clin Oncol.

[55] Zauderer MG, Krug LM (2012). Novel therapies in phase II and III trials for malignant pleural mesothelioma. J Natl Compr Canc Netw.

[56] Thatcher N, Chang A, Parikh P, Rodrigues Pereira J, Ciuleanu T, von Pawel J, Thongprasert S, Tan EH, Pemberton K, Archer V, Carroll K (2005). Gefitinib plus best supportive care in previously treated patients with refractory advanced non-small-cell lung cancer: results from a randomised, placebo-controlled, multicentre study. Lancet.

[57] Mok TS, Wu YL, Thongprasert S, Yang CH, Chu DT, Saijo N, Sunpaweravong P, Han B, Margono B, Ichinose Y, Nishiwaki Y, Ohe Y, Yang JJ (2009). Gefitinib or carboplatin-paclitaxel in pulmonary adenocarcinoma. N Engl J Med.

[58] Rosell R, Carcereny E, Gervais R, Vergnenegre A, Massuti B, Felip E, Palmero R, Garcia-Gomez R, Pallares C, Sanchez JM, Porta R, Cobo M, Garrido P (2012). Erlotinib versus standard chemotherapy as first-line treatment for European patients with advanced EGFR mutation-positive non-small-cell lung cancer (EURTAC): a multicentre, open-label, randomised phase 3 trial. Lancet Oncol.

[59] Landreville S, Agapova OA, Matatall KA, Kneass ZT, Onken MD, Lee RS, Bowcock AM, Harbour JW (2012). Histone deacetylase inhibitors induce growth arrest and differentiation in uveal melanoma. Clin Cancer Res.

[60] Moss AC, Jacobson GM, Walker LE, Blake NW, Marshall E, Coulson JM (2009). SCG3 transcript in peripheral blood is a prognostic biomarker for REST-deficient small cell lung cancer. Clin Cancer Res.

